# Knocking out genes to reveal drivers of natural selection on phenotypic traits: a study of the fitness consequences of albinism

**DOI:** 10.1098/rspb.2025.1458

**Published:** 2025-08-27

**Authors:** Alexander T. Funk, Jan Martin, Michael Clark, Antoine Païta, Chris J. Jolly, Richard Shine

**Affiliations:** ^1^ School of Natural Sciences, Macquarie University, Sydney, New South Wales, Australia; ^2^ Independent Researcher, Germany; ^3^ Applied BioSciences, Macquarie University, Sydney, New South Wales, Australia; ^4^ ENS de Lyon Département de Biologie, Lyon, Auvergne-Rhône-Alpes, France; ^5^ Research Institute for the Environment and Livelihoods, Charles Darwin University, Darwin, Northern Territory, Australia

**Keywords:** Bufo marinus, coloration, genetic engineering, intraspecific competition

## Abstract

Conclusions about the adaptive significance of phenotypic traits typically rely on correlations between the trait and fitness, but pleiotropic effects of a single trait on fitness and covariation among traits can confound such comparisons. For example, a trait may have several benefits or costs which may be affected by its correlation to some other trait. We overcame this barrier by using CRISPR-Cas9 in captive cane toads (*Rhinella marina*) to create gene-knockout albinos. Using this approach, we could evaluate direct effects of a single allele on fitness by comparing rates of survival, growth and development of albino versus pigmented siblings. Contrary to the prevailing view that albinism is rare solely due to reduced crypsis (increased vulnerability to predation), we found that albino tadpoles and terrestrial-phase toads were competitively inferior to their pigmented siblings even in the absence of predation. Visual impairment appears to explain this cost in the terrestrial life-stage, as albino toads had lower foraging success, were less accurate when striking at prey, and needed higher light levels to forage successfully. Our findings suggest that competitive inferiority may contribute to selection against albinism in the wild and demonstrate the utility of gene knockouts for experimental evolutionary biology.

## 1. Introduction

Gene editing using CRISPR-Cas9 [1,2] offers a promising new approach for testing the fitness consequences of phenotypes. Using CRISPR, researchers can inactivate specific genes [3,4] to assess their effects on fitness in varied ecological and evolutionary contexts [5]. This method eliminates a potential problem with assessing fitness correlates of phenotypic variation. If a novel trait arises, natural selection can promote or suppress its spread through the population through various pathways. Often it is difficult to disentangle the effects of multiple drivers of selection acting in concert. This problem is especially pronounced when selection for a trait favours its association with other traits. For example, a trait that reduces an individual’s vulnerability to predation might enable that individual to be active over a wider range of times and places because it can now safely access resources in situations that were previously too risky. In that situation, we might expect the correlated evolution of traits that affect foraging behaviour and the genes that enable the novel antipredator ability. When selection for one trait is correlated with the evolution of others, simply comparing conspecifics that differ in the putative trait of interest is confounded with variation in its correlates. Experimentally knocking out a single gene removes that confounding effect and allows us to distinguish between multiple drivers of selection.

Individuals with experimentally inactivated genes (hereafter referred to as gene knockouts) are widely used by medical (e.g. [6–8]) and pest control researchers (e.g. [9–11]), developmental biologists (e.g. [12–14]) and immunologists (e.g. [15–17]), but have rarely been used to address ecological and evolutionary questions (e.g. [18]). Using gene knockouts to test the effects of specific phenotypes on fitness could further understanding of the drivers of phenotypic variation in wild populations. To demonstrate the value of gene knockouts for investigations of eco-evolutionary hypotheses, we created *tyrosinase* knockout cane toads (*Rhinella marina*) and used them to examine potential drivers of selection against albinism.

An individual’s colour can have major effects on its fitness, resulting in intense selection. Colour patterns mediate predator–prey dynamics by providing crypsis [19–21], signalling aposematism [19,20,22,23] or enhancing mimicry systems [19,24,25]. Colour is also important in intraspecific communication [20,26] and in physiological processes such as thermoregulation [27,28] and resistance to ultraviolet radiation [29,30]. Because colouration can affect fitness via multiple pathways, the adaptive significance of colour polymorphisms within populations has been the focus of much research and debate (e.g. [31–35]). Some chromatic polymorphisms are thought to be maintained by sexual selection [36–38], others by heterozygote advantage [39,40] and yet others by frequency-dependent selection [41–43]. One colour phase that seems to be consistently selected against across taxa but continues to arise via mutations is albinism [44–46].

The term ‘albinism’ is used to refer to conditions that include some form of loss of melanin [47–49]. Albinism can arise via numerous genetic pathways; for example, mutations in 12 genes are known to cause albinism in humans [48,50]. Oculocutaneous albinism type 1 (often referred to as ‘true albinism’) is characterized by complete absence of melanin in the skin, hair and eyes [48,50]. This form of albinism is familiar to the public due to its occurrence in humans, livestock and pets and is caused by mutations in the gene responsible for production of *tyrosinase* (an enzyme necessary for melanin formation; [48,50,51]). Although true albinism (hereafter referred to as albinism) has been documented in many vertebrate species, it rarely occurs at high frequencies in the wild, which suggests that albinism confers a selective disadvantage [52].

The most widely cited putative driver of selection against albinism is its effect on crypsis: albinos are often more conspicuous than pigmented conspecifics and therefore may be more vulnerable to predation. Despite the dominance of this hypothesis in public discourse and scientific literature, empirical evidence is scarce. Some experiments reported higher rates of avian predation on white versus coloured mice [53–55], whereas a study using plaster models of snakes found no effect of simulated albinism on rates of attack by birds [56]. Although the possibility of increased vulnerability to predation is often mentioned in taxon-specific field reports of albinism (e.g. [57–59]), it has rarely been tested directly. No studies have directly tested alternate mechanisms by which albinos could be selected against, although effects on social behaviour [60,61], genetically linked developmental abnormalities [57,62] and foraging success [63] have been suggested and others, including effects on competitive ability, are plausible. Research in humans and model organisms has also shown that albinism can affect vision [64,65], UV resistance [48] and immune function [66,67], but the fitness ramifications of these effects have not been assessed. The scarcity of such studies reflects the difficulty of obtaining large numbers of albino individuals in non-model organisms. Traditional approaches would require researchers to capture, rear and breed wild-caught albinos to achieve robust experimental designs, as the effects of albinism are often confounded by relatedness of albinos to one another versus pigmented individuals when albinos are discovered *in situ*. However, the advent of efficient, cost-effective gene editing technology now makes these experimental challenges surmountable.

Cane toads offer an ideal opportunity to test the effects of albinism on competitive ability across two life stages that exhibit different behaviours and foraging modes. Cane toad tadpoles form schools [68] and rely on chemical cues while foraging [69], whereas terrestrial-phase toads are less gregarious and rely on visual cues to capture prey [70,71]. We hypothesized that albino cane toads may be competitively inferior to their pigmented kin during both life stages. The competitive disadvantage of albino tadpoles may be induced by stress associated with the presence of pigmented individuals, as suggested by behavioural alterations observed in albino fish [60,61,72]. In toads, vision-related deficits may render albinos ineffective foragers relative to pigmented individuals, as albinism is associated with reduced visual acuity and impaired stereoscopic vision [64,65]. To assess effects of albinism on competitive ability, we reared F1 albino gene knockouts and pigmented siblings in same-phenotype and mixed-phenotype treatments and compared rates of growth and survival between treatments and phenotypes. We then conducted behavioural trials during the terrestrial life-stage to link variation in survival and growth to effects of albinism on vision. Our study is the first to use CRISPR gene knockouts to experimentally test the fitness consequences of an individual phenotypic trait in a non-model vertebrate, demonstrating the powerful potential of this approach for evolutionary and ecological research.

## 2. Methods

### (a) Obtaining eggs

**Figure 1.**
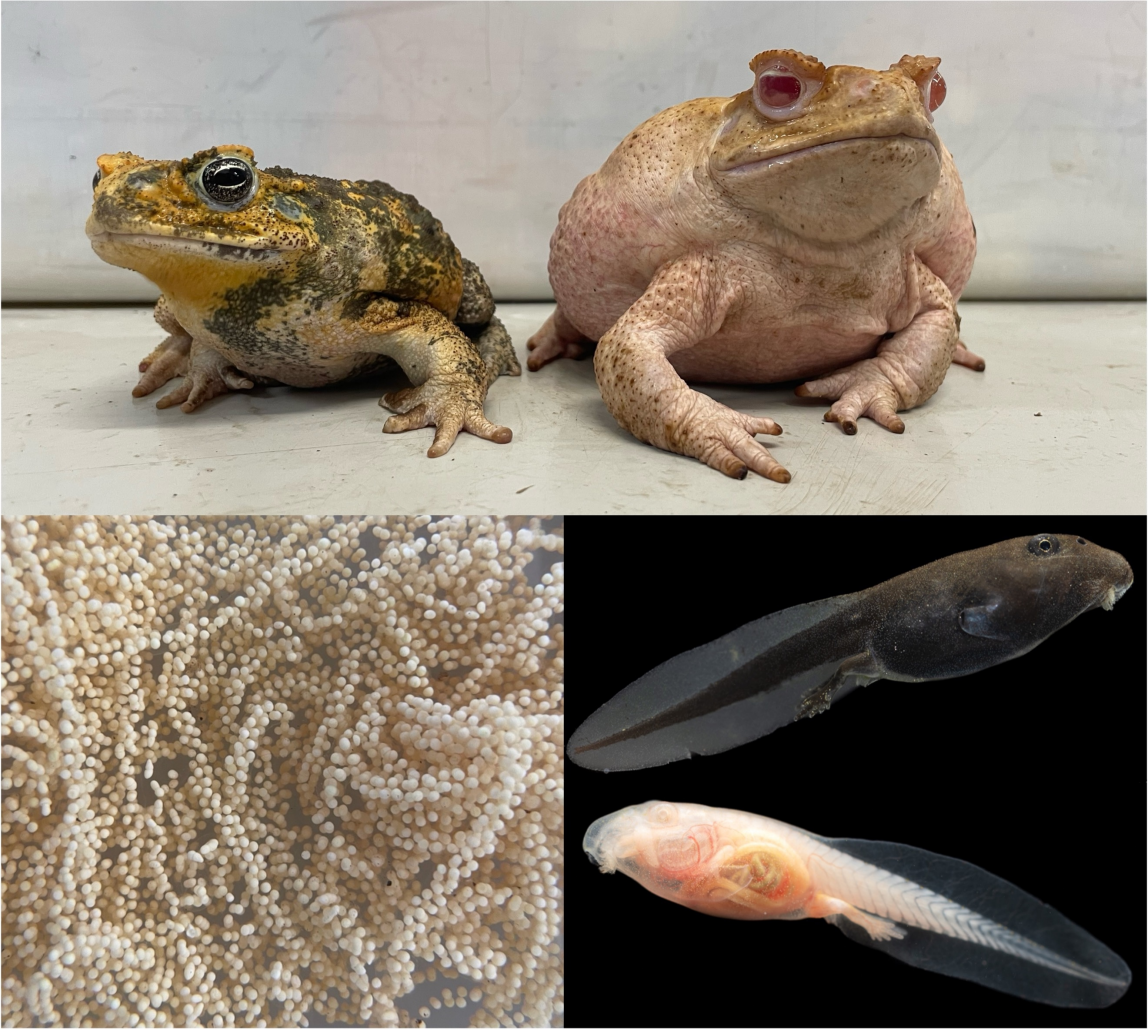
Top: Male and female mosaic albino cane toads (*Rhinella marina*) produced via CRISPR-knockout of *tyrosinase* in early developmental zygotes. Bottom left: albino eggs laid after hormone-induced breeding of mosaic albino cane toads. Bottom right: sibling pigmented and albino tadpoles (photo by Etienne Littlefair).

We bred one pair of adult F0 edited albino toads produced via CRISPR injections targeting *tyrosinase* [73] to obtain the clutch of eggs used in this experiment (figure 1). Although several genes could be disrupted to induce phenotypic albinism, we chose to knock out *tyrosinase* because the mutation is recessive, which allows heterozygote carriers to maintain normal function, and because *tyrosinase*-deficiency is the most well-characterized form of albinism, enabling more robust comparison with existing literature. We housed the toads for approximately 12 hours in a 75 l tub containing a shallow layer of bore water and administered a subcutaneous injection of leuprorelin acetate (Lucrin; Abbott Australasia, Kurnell, Australia) diluted in amphibian Ringer’s solution (concentration = 0.25 mg ml^−1^) to stimulate breeding. We injected the male toad with 0.25 ml of solution and the female with 0.75 ml and allowed the pair to spawn overnight. The following morning, we collected all eggs.

### (b) Genotyping and husbandry of prefeeding larvae

Following Clark *et al*. [73], we determined the genotype of F1 offspring based on pigmentation patterns at specific developmental stages. Maternal genotype contribution was assessed at 0−1 hours post-fertilization, when egg pigmentation reflects the maternal genotype through either active melanin synthesis during oogenesis or maternally contributed proteins and mRNA. Paternal transmission was evaluated at 4 days post-fertilization (i.e. around Gosner stage 25 [74]), when zygotic gene expression allows functional paternal alleles to rescue the albino phenotype. All eggs were albino, reflecting maternally inherited null *tyrosinase* alleles. Larvae developing pigmentation at 4 days post-fertilization had inherited functional paternal *tyrosinase* alleles and were classified as heterozygous. These pigmented, phenotypically wild-type heterozygotes served as controls throughout the study. Because of this genotyping approach, we reared all larvae communally in 20 l of bore water until they could be visually phenotyped as either pigmented or albino.

Of the tadpoles that survived to the free-swimming stage, 383 (76.9%) were pigmented (i.e. heterozygous for the null *tyrosinase* allele, with dark pigmentation in both the body and eyes) and 115 (23.1%) were true albinos (i.e. homozygous for the null *tyrosinase* allele, lacking dark pigmentation in both the body and eyes). Heterozygote carriers for *tyrosinase* mutations in other species are phenotypically wild-type [75,76], making them suitable for our assessment of potential costs of the albino phenotype. While we cannot entirely exclude the possibility of heterozygote effects, at most they would have reduced the magnitude of the differences observed between phenotypes.

### (c) Growth, development and survival of tadpoles

To assess whether albinism affected rates of growth, development and survival during the tadpole stage, we randomly allocated 216 tadpoles (108 albino and 108 pigmented) to 13 l experimental tubs filled with bore water. We assigned each tub to one of two competition treatments; tadpoles were either reared in tubs containing only individuals of the same phenotype (same-phenotype treatment) or in tubs in which 50% of individuals were albino and 50% were pigmented (mixed-phenotype treatment). We assigned 12 tadpoles to each of 18 tubs (*n* = 6 albino same-phenotype, *n* = 6 pigmented same-phenotype, and *n* = 6 mixed-phenotype replicates) and checked each tub daily for dead tadpoles. We fed tadpoles a 3 : 1 ratio of ground rabbit pellets (Vetafarm, Wagga Wagga, Australia) to tropical fish flakes (Petbarn, Sydney, Australia) and provided each tub with *per capita* rations equivalent to 10 mg per tadpole per day, which is sufficient for rearing cane toad tadpoles to metamorphosis but allows for competition between individuals. We checked tubs for deaths and metamorphosed individuals prior to each daily feeding and adjusted rations when either was detected to ensure that *per capita* rations remained at 10 mg per tadpole per day for the duration of the experiment. To ensure optimal water quality, we replaced two-thirds of the water in each tub with fresh bore water every fifth day.

On days 5 and 10 of the experiment, we removed all surviving tadpoles from their experimental tubs, Gosner-staged and weighed each one to the nearest milligram and then returned them to their tubs. When tadpoles metamorphosed (i.e. when all four limbs had emerged), we recorded the date of metamorphosis, collected them, and moved them into a transitional tub consisting of 50% land and 50% water and corresponding to their phenotype and competition treatment. Because a small proportion of tadpoles in some clutches remain tadpoles indefinitely, it is standard practice to impose an end-date for growth experiments as opposed to waiting for all individuals to metamorphose (e.g. [77,78]). Here, we continued to record survival data and time to metamorphosis until no metamorph had been detected in any tub for 14 days. We considered the 20 remaining tadpoles to have survived but excluded them from analyses of time to metamorphosis.

### (d) Rates of growth and survival of terrestrial-phase toads

To assess whether albinism affected rates of growth and survival during the terrestrial stage, we individually allocated 108 metamorph toads (54 albino and 54 pigmented) from the tadpole experiment to experimental enclosures assigned to one of two competition treatments (same-phenotype or mixed-phenotype). Prior to sorting toads into competition treatments (i.e. for one week after the first metamorph was detected), we provided toads in communal transitional tubs with an unlimited supply of live springtails. On day 7, we weighed the toads, measured their snout-urostyle lengths (SULs) and individually toe-clipped them for identification. We then assigned toads to one of three replicate experimental enclosures corresponding to their phenotype and the competition treatment to which they were assigned during the tadpole stage. We stratified toads by body size to minimize initial size variation in each experimental enclosure and housed 12 toads in each of the nine enclosures (*n* = 3 albino same-phenotype, *n* = 3 pigmented same-phenotype and *n* = 3 mixed-phenotype enclosures).

Each day, we supplied toads in experimental enclosures with live termites. To standardize and limit food availability among enclosures, the total mass of termites added to an enclosure corresponded to the total mass of toads in that enclosure. We provided each enclosure with 0.155 mg of termites per milligram of toad mass (see electronic supplementary material for a description of how this ratio was determined). Every fifth day, we removed all surviving toads from the experimental enclosures, individually identified them from toe clips, weighed them and measured their SULs. We then adjusted enclosure-specific food rations to account for changes in total toad mass due to growth and/or deaths over the preceding five days.

We kept toads in these experimental enclosures for 15 days before re-sorting them to assess whether effects of albinism on growth and survival were influenced by the stage at which albino toads encountered pigmented conspecifics. On day 15, we removed 59 of the remaining toads from the albino and pigmented same-phenotype enclosures (*n* = 29 albino, *n* = 30 pigmented), size-matched them and constructed three new mixed-phenotype enclosures, each containing six pigmented and six albino toads. We also constructed two new same-phenotype enclosures (one per phenotype) and stocked them with the 11 remaining albino and 12 remaining pigmented toads, respectively. We then collected data on growth and survival for an additional 20 days.

### (e) Behaviour of toads

To assess whether foraging success differed between albino and pigmented toads, we conducted two behavioural trials. We designed the first trials to assess toads’ ability to capture prey under different light conditions. During these trials, we housed toads individually in shallow black circular containers (11 cm diameter, 3 cm height) with clear lids and exposed each toad to 20 live termites in either ambient light, dim light or complete darkness. Although we measured light intensity (in lux) for each of these light treatments, we do not report the values that we obtained here because our measurement tool was not precise enough to allow for exact replication of our light conditions from reported lux values. We instead describe how we achieved those light conditions to facilitate replication of our experimental treatments. For the ambient light treatment, we placed experimental containers on a benchtop in the laboratory with overhead lights illuminated and all windows uncovered. We created dim light conditions by placing experimental containers in a large black rectangular tub (65 L × 42 W × 27 D cm) on the same benchtop under the same light conditions and covering the top of the tub with four layers of 70% shade cloth. For the trials conducted in complete darkness, we placed the experimental containers on the same benchtop under the same light conditions but housed them beneath an upside-down black tub. We allowed toads to acclimate to experimental containers for 3 minutes before adding 20 live termites to each tub. We then allowed toads to forage for 7 minutes before removing them from experimental containers and counting the number of termites remaining in each container. One trial was conducted per toad per day in light, dim or dark conditions. We tested all toads under all light conditions and randomized the order in which each toad was exposed to each light treatment over the three days during which trials were conducted.

Following light-intensity trials, we conducted direct observation trials to assess whether albino and pigmented toads differed in their ability to locate and capture prey. We housed toads individually in the same experimental containers used during light trials, allowed them to acclimate for three minutes and then exposed each toad to 20 termites in ambient light conditions while a single observer watched. The observer first recorded the time at which each toad first struck at a termite. If a toad did not strike within 2 minutes, we concluded the trial and excluded that individual from analyses. We allowed all individuals that struck within 2 minutes to feed for an additional 5 minutes, and the observer recorded the total number of times that each individual struck. After 5 minutes, we removed toads from experimental containers and recorded the total number of termites remaining in the containers. From these data, we calculated the strike accuracy of each toad ($\frac{termites\;consumed}{number\;of\;strikes}$ * 100) and its time to first strike. We conducted trials over 2 days and used the same observer for all trials to standardize potential observer bias.

### (f) Statistical analyses

We performed all statistical analyses using R version 4.4.3 [79]. We created general and generalized linear models using the ‘lm’ and ‘glm’ functions in base R and constructed general and generalized linear mixed-effects models using the ‘lmer’ and ‘glmer’ functions in the package *lme4* version 1.1−36 [80] and the ‘glmer.nb’ function in the package *MASS* version 7.3−64 [81]. We assessed predictor significance for each model using the ‘Anova’ function in the package *car* version 3.1−3 [82].

#### (i) Rates of growth, development and survival of tadpoles

To assess potential interactive effects of phenotype and competition treatment on rates of growth (measured as change in mass) and development (measured as change in Gosner stage), we used general linear models. Because we could not individually identify tadpoles in each tub, we first calculated date- and phenotype-specific mean masses and Gosner stages of tadpoles in each tub. We then subtracted the mean values of our day 5 measurements from those of our day 10 measurements to quantify the change in mean mass and Gosner stage of tadpoles of each phenotype in each tub. We used these values (Δmass and Δstage, respectively) as response variables in two separate general linear models and assessed predictor significance using analysis of variance tests.

To assess interactive effects of phenotype and competition treatment on time to metamorphosis (measured in days), we used a negative-binomially distributed generalized linear model. We then used a binomially distributed generalized linear model to assess potential interactive effects of phenotype and competition treatment on tadpole survival. We considered tadpoles to have survived the larval stage if they either metamorphosed or were alive when we concluded the tadpole phase of the experiment (i.e. after no metamorph had been detected in any replicate for 14 days). We assessed predictor significance for both models using likelihood-ratio chi-squared tests.

#### (ii) Growth and survival of terrestrial-phase toads

Because surviving toads from same-phenotype replicates were subsampled and assigned to new competition treatments on day 15, we conducted all survival and growth rate analyses separately for days 0−15 and days 15−35 of the experiment. We first assessed the effect of initial size (measured as SUL) and interactive effects of phenotype and competition treatment on toad growth (measured as change in SUL). Only toads that survived for the duration of each phase of the experiment were included in growth analyses. We calculated change in toad SUL by subtracting the initial measures of SUL from the final measures of SUL during each phase. We then used this value (ΔSUL) as the response variable in general linear mixed-effects models and assessed predictor significance using chi-squared tests. Replicate ID was included as a random effect in both growth rate models to account for variation in growth rates among replicate enclosures. To assess the effect of initial size and interactive effects of phenotype and competition treatment on toad survival during each phase of the experiment, we used binomially distributed generalized linear models and assessed predictor significance using likelihood-ratio chi-squared tests.

#### (iii) Behaviour of toads

We used a negative-binomially distributed generalized linear mixed model to assess the effect of toad size and interactive effects of light treatment and phenotype on foraging success (measured as number of termites consumed in seven minutes) during light trials. We assessed predictor significance using a chi-squared test and included toad ID as a random effect to account for repeated measures. We then used a negative-binomially distributed generalized linear model to investigate the effect of phenotype on time to prey recognition (measured as seconds until first strike) during observation trials. To assess the effect of phenotype on strike accuracy (measured as proportion of strikes that were successful), we used a binomially distributed generalized linear model. We assessed predictor significance for both models using likelihood-ratio chi-squared tests.

## 3. Results

### (a) Rates of growth and development of tadpoles

**Figure 2.**
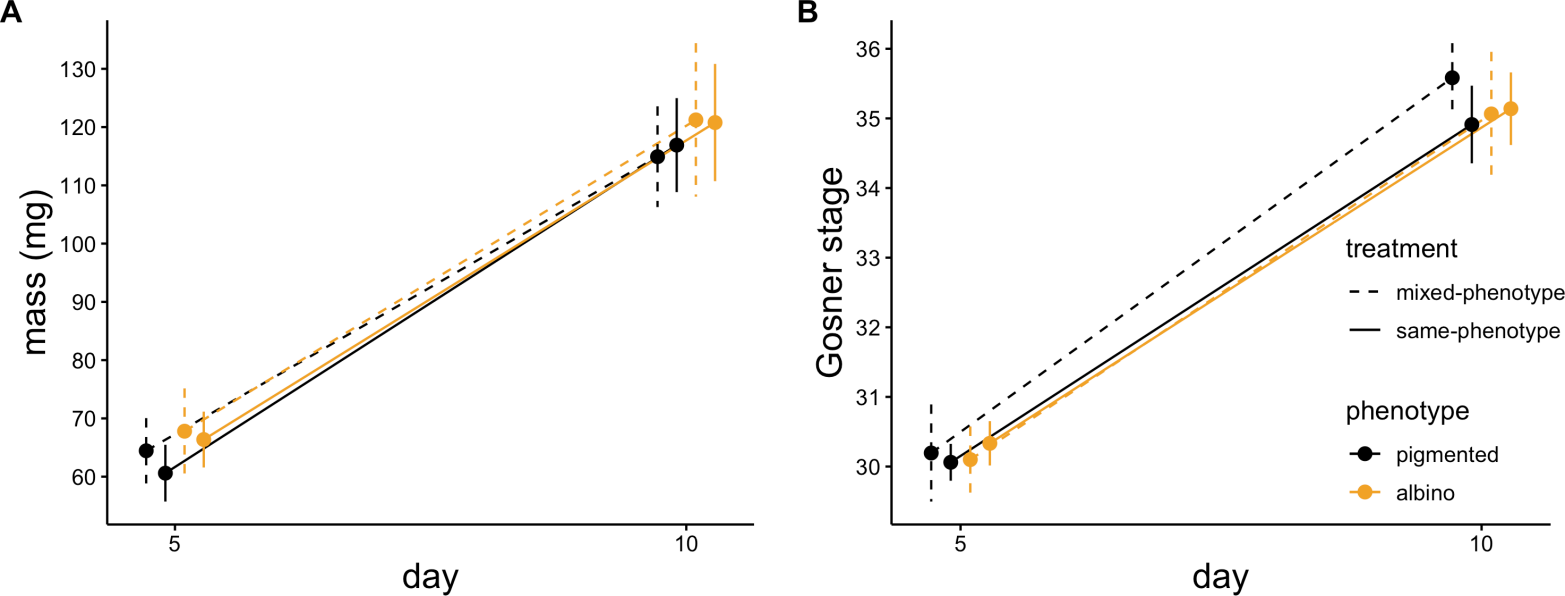
(A) Average masses and (B) Gosner stages of albino and pigmented tadpoles reared in mixed-phenotype and same-phenotype tubs. Measurements were taken on days 5 and 10 of the experiment. Error bars represent 95% confidence intervals bounding means.

We detected no significant difference in mean growth rates of tadpoles (Δmass) between phenotypes (*F* = 0.003, *p* = 0.954) or competition treatments (*F* = 0.85, *p* = 0.368) and no significant interactive effect of phenotype and competition treatment on growth rates (*F* = 0.17, *p* = 0.683; figure 2A). Mean rates of tadpole development (Δstage) did not differ significantly between phenotypes (*F* = 0.97, *p* = 0.338) or competition treatments (*F* = 2.11, *p* = 0.162), and we detected no significant interactive effect of phenotype and competition treatment on rates of development (*F* = 0.63, *p* = 0.438; figure 2B).

### (b) Tadpole survival and time to metamorphosis

**Figure 3.**
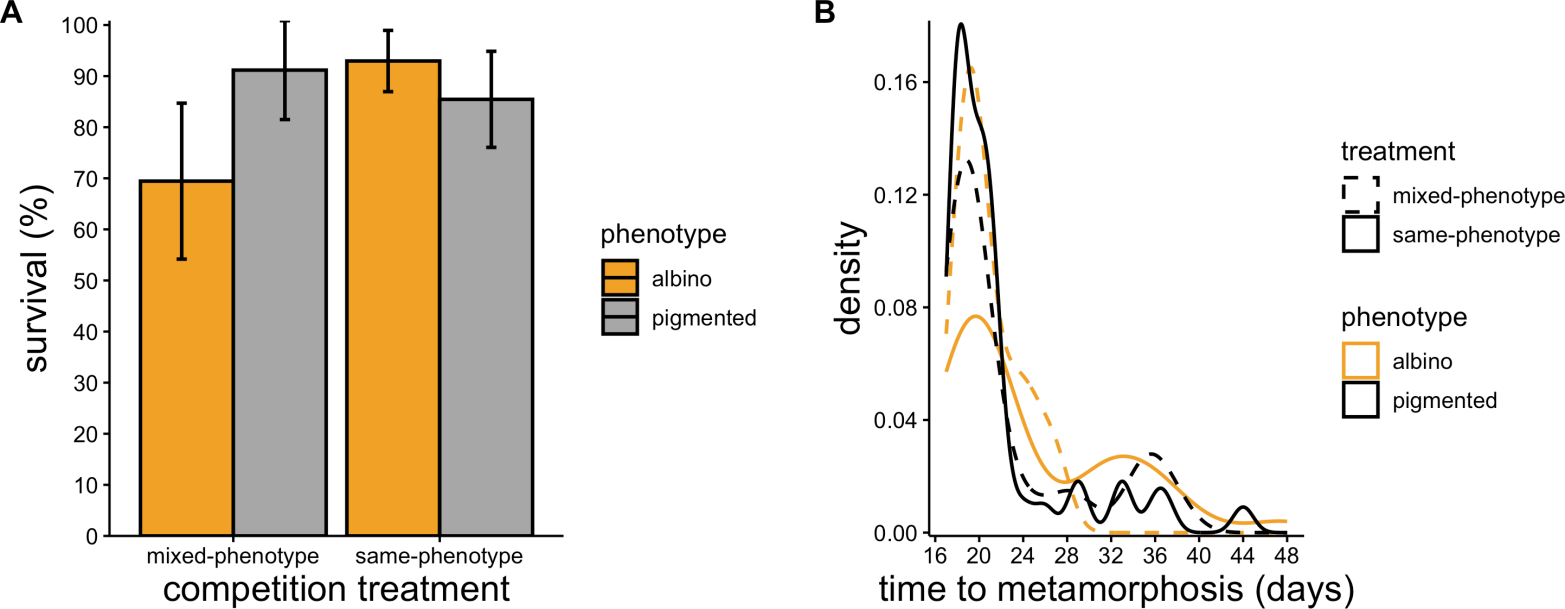
(A) Percentage survival ± 95% confidence intervals and (B) distribution of days to metamorphosis of albino and pigmented tadpoles reared in mixed-phenotype and same-phenotype tubs. Tubs were checked for dead tadpoles and metamorphs daily for the duration of the experiment.

Probability of survival through the tadpole stage was influenced by the interaction between phenotype and competition treatment (LR *χ*^2^ = 6.93, *p* = 0.008; figure 3A). This interactive effect was driven by reduced survival of albino tadpoles in the mixed-phenotype treatment relative to those in the same-phenotype treatment (figure 3A). The interaction between phenotype and competition treatment also affected time to metamorphosis (LR *χ*^2^ = 5.03, *p* = 0.025; figure 3B); albino tadpoles metamorphosed earlier in the mixed-phenotype treatment (x̄_days_ = 20.9, CI = 19.8−21.9; figure 3B) than in the same-phenotype treatment (x̄_days_ = 24.7, CI = 22.8−26.6; figure 3B).

### (c) Rates of growth and survival of terrestrial-phase toads

**Figure 4.**
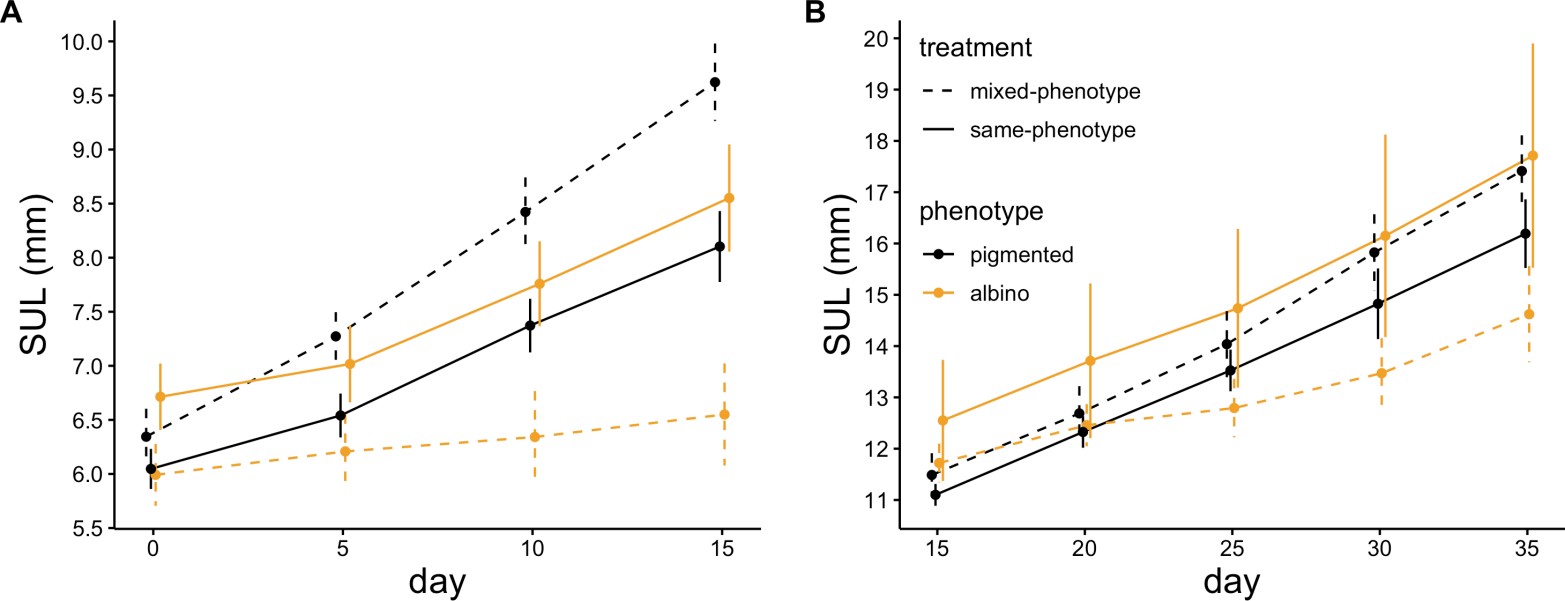
Average snout–urostyle lengths (SULs) ± 95% confidence intervals of albino and wild-type toads reared in mixed-phenotype and same-phenotype enclosures. Toads were measured at 5 day intervals for the duration of the 35 day experiment. Averages from (A) days 0−15 and (B) days 15−35 are depicted separately because toads assigned to same-phenotype competition treatments from days 0−15 were subsampled and used to create new experimental treatments on day 15 to assess whether time of first contact with individuals of a different phenotype affected growth and survival.

Mean growth rates of individual toads, measured as change in SUL, during the first 15 days of the experiment were influenced by the interaction between phenotype and competition treatment (*χ*^2^ = 37.03, *p* < 0.001; figure 4A) but not by initial SUL (*χ*^2^ = 0.47, *p* = 0.491). After re-sorting and size matching, growth rates during the last 20 days of the experiment were influenced by both initial SUL (*χ*^2^ = 8.53, *p* = 0.003; figure 4B) and the interaction between phenotype and competition treatment (*χ*^2^ = 11.34, *p* < 0.001; figure 4B). During both phases of the experiment, growth rates of albino and pigmented toads in the same-phenotype treatment were similar, whereas growth rates of albino toads in mixed-phenotype treatments were lower than those of all other groups and growth rates of pigmented toads in mixed-phenotype treatments were higher than those of all other groups (figure 4A,B).

**Figure 5.**
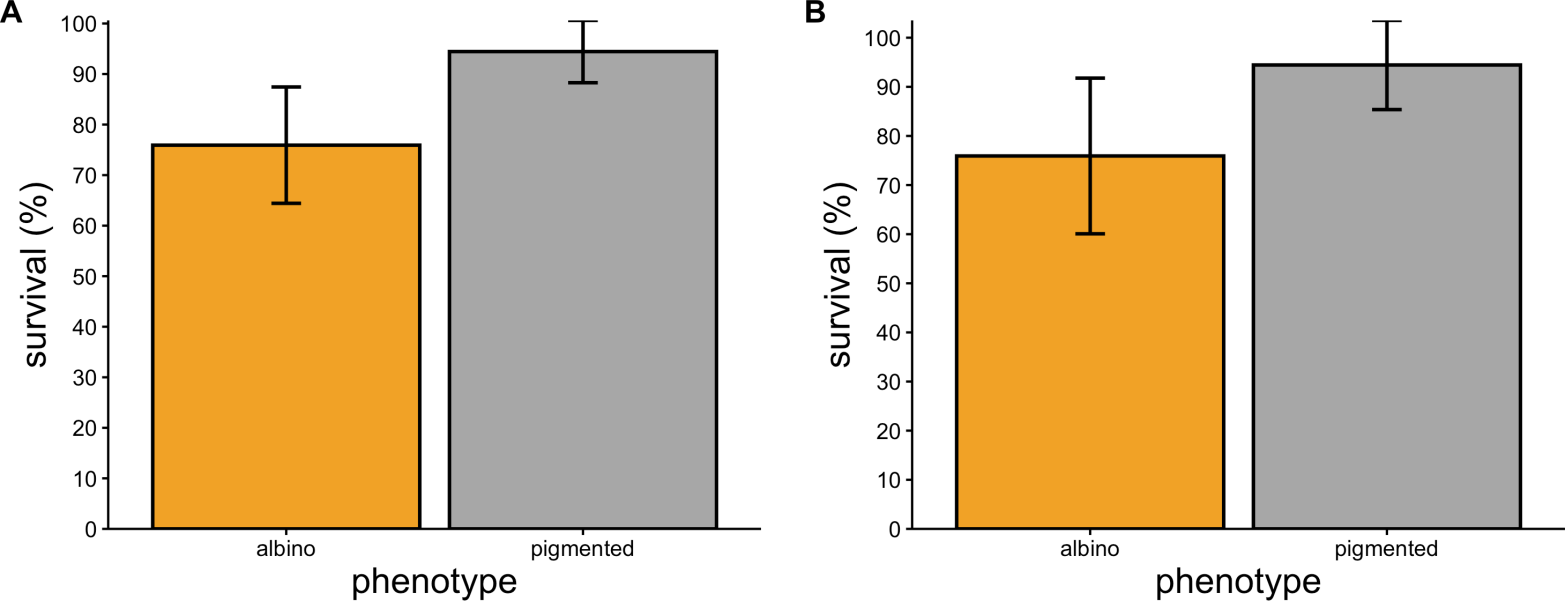
Percentage survival ± 95% confidence intervals of albino and pigmented toads. Survival rates are depicted separately for (A) days 0−15 and (B) days 15−35 of the 35 day experiment because toads assigned to same-phenotype competition treatments from days 0−15 were subsampled and used to create new experimental treatments on day 15 to assess whether time of first contact with individuals of a different phenotype affected growth and survival. No significant effect of competition treatment on survival was detected. Therefore, competition treatment was not included as a factor in these visualizations.

Probability of survival through the first 15 days of the experiment was influenced by initial size (LR *χ*^2^ = 12.43, *p* < 0.001) and phenotype (LR *χ*^2^ = 8.03, *p* = 0.004), and we detected no significant effect of treatment (LR *χ*^2^ = 0.61, *p* = 0.433) or interaction between phenotype and treatment (LR *χ*^2^ = 1.22, *p* = 0.268) on probability of survival. Smaller toads were more likely to die during this phase, as were albinos (figure 5A). Probability of survival through the last 20 days of the experiment (after re-sorting and size matching) was associated with initial size (LR *χ*^2^ = 26.31, *p* < 0.001), and we detected no significant effect of phenotype (LR *χ*^2^ = 2.23, *p* = 0.136), treatment (LR *χ*^2^ = 2.18, *p* = 0.140), or their interaction (LR *χ*^2^ = 0.152, *p* = 0.697) on a toad’s probability of survival (figure 5B).

### (d) Behaviour of toads

**Figure 6.**
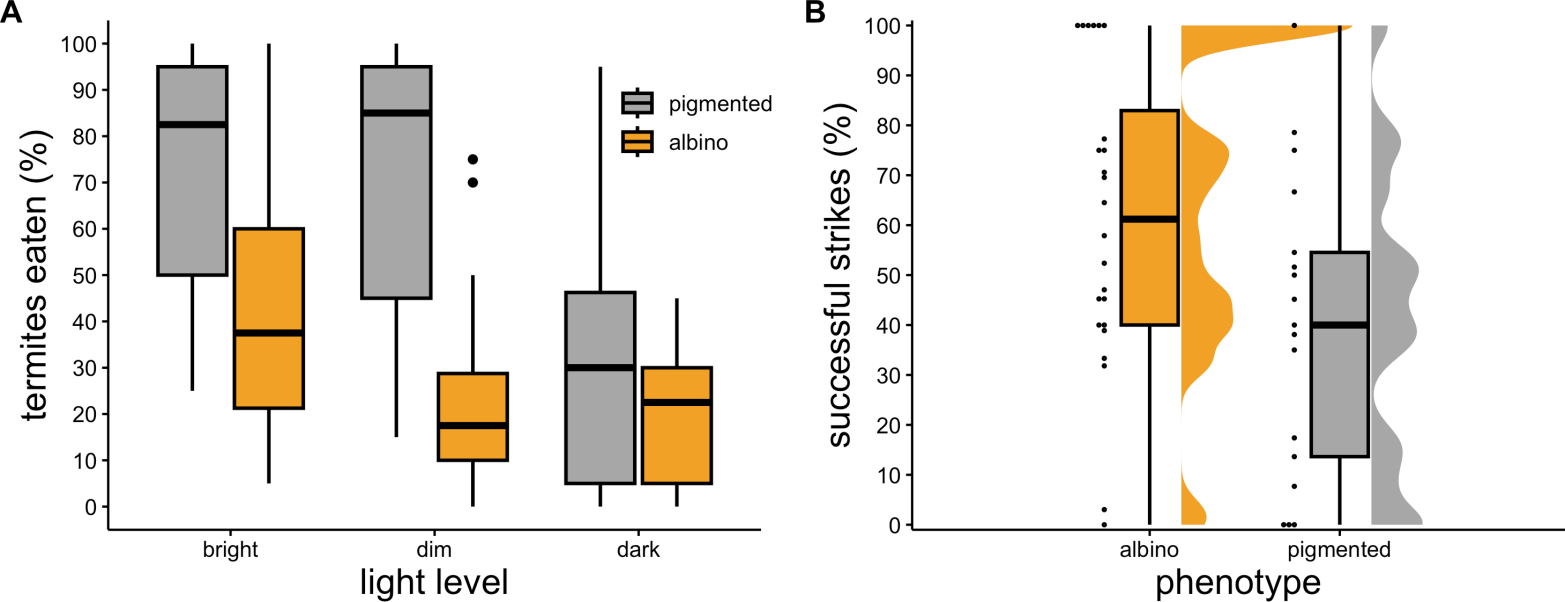
(A) Foraging success (measured as percentage of termites consumed) of albino and pigmented toads exposed to termites in bright, dim and dark conditions. (B) Strike accuracy (measured as percentage of strikes that were successful) of albino and pigmented toads in ambient light. We assessed the foraging success of each toad in all light conditions, and a single observer scored all trials assessing strike accuracy in ambient light.

During light trials, foraging success of toads was influenced by body size (*χ*^2^ = 10.38, *p* = 0.001) and the interaction between phenotype and light treatment (*χ*^2^ = 9.63, *p* = 0.008; figure 6A). Pigmented toads were more successful foragers than were albinos in all light conditions, and foraging success of pigmented toads was high in bright and dim light but low in total darkness (figure 6A). Albino toads were best at foraging in bright light and had low success in both dim light and total darkness (figure 6A). During observation trials, time to prey recognition (i.e. first strike) did not differ significantly between phenotypes (LR *χ*^2^ = 2.79, *p* = 0.095), but the strikes of pigmented toads were more likely to be successful than were those of albinos (LR *χ*^2^ = 4.23, *p* = 0.039; figure 6B).

## 4. Discussion

Although several mechanisms of selection against albinism have been proposed (e.g. [57,60–62]), elevated predation risk has generally been suggested as the primary force suppressing albinism in wild populations. Nonetheless, few studies have critically assessed this assumption, and those that have found conflicting patterns; in some, albinism has no effect (e.g. [56]), while in others, it increased predation risk (e.g. [53–55,57]). The lack of research is unsurprising, as wild albinos are rarely encountered in large numbers and comparative studies of naturally occurring albino and pigmented individuals are often confounded by relatedness of albinos versus pigmented conspecifics. Using cane toads as a model, we overcame these challenges by using CRISPR-Cas9 to knockout the gene that encodes *tyrosinase* (an enzyme critical for melanin synthesis; [51]). This allowed us to test an alternative explanation for the rarity of albinos; that albinism reduces competitive ability. Our use of F1 knockout siblings allowed for a powerful comparison of the competitive ability of albino and pigmented toads, with no confounding clutch effects and a uniform likelihood of off-target effects of CRISPR editing. Our findings suggest that albino toads are competitively inferior to pigmented individuals as both aquatic tadpoles and terrestrial toads.

During the tadpole stage, competition between phenotypes affected the survival probability and larval duration of albino tadpoles. Albinos in the mixed-phenotype treatment were less likely to survive and metamorphosed sooner than did albinos in the same-phenotype treatment. Despite these effects, competition treatment, tadpole phenotype, and their interaction did not significantly influence rates of larval growth or development earlier in the larval period. These findings suggest that albinos reared with pigmented kin were disadvantaged by some competitive effect not directly related to foraging success. Reduced survival and early metamorphosis might be explained by stress and behavioural alteration of albinos associated with the presence of pigmented kin, as facultative reduction of the larval period often is associated with environmental stressors in amphibians [83–85] and albinism influences stress responses and social behaviour in fish [60,61,72,86,87]. Because we did not assay tadpole behaviour, future behavioural studies are needed to clarify mechanisms underlying the apparent competitive inferiority of albino tadpoles. Such studies should assess behavioural responses of albinos to albino versus pigmented tadpoles along with other factors known to influence behaviour of albinos (e.g. bright light [88]).

During the terrestrial life-history stage, competition between phenotypes affected growth rates. Growth rates did not differ significantly between albinos and pigmented toads raised with siblings of the same phenotype, but albino toads grew more slowly, and pigmented toads more quickly, in the mixed-phenotype treatment than the same-phenotype treatment. Thus, growth of albinos appears to be inhibited by the presence of pigmented competitors. This effect weakened but was still present when albinos were exposed to competition with pigmented conspecifics later in development, suggesting that competition with pigmented individuals during early development (i.e. as tadpoles and toadlets) may have carry-over effects on growth rates. Survival rates also differed between albino and pigmented toads. Albino toads were less likely to survive the first 15 days of the experiment than were pigmented toads, regardless of competition treatment, suggesting that albinos had lower fitness even when raised without competition from pigmented kin.

Although off-target effects of CRISPR modification [89,90] could potentially explain the reduced survival and competitive inferiority of our albino toads, such pronounced effects of off-target edits are unlikely [91]. We favour an alternative hypothesis; that the effects of albinism on vision best explain the differences in survival and competitive ability between albinos and pigmented individuals. Albino vertebrates have less visual acuity and reduced stereoscopic vision relative to pigmented conspecifics [64,65], and these visual-system impairments could impact foraging success in toads, which are primarily visual predators [70,71]. Some individuals may starve due to an inability to locate and capture prey, which would explain our observation of reduced survival of albinos regardless of competition treatment. Others may be able to capture prey but do so less efficiently than pigmented individuals with intact vision, leading to the reduced growth rates we observed in albinos that competed with pigmented kin. Following our competition experiments, we tested this hypothesis by assessing effects of light levels on foraging success of albinos versus pigmented individuals and comparing strike accuracy of albino versus pigmented toads.

In those behavioural trials, both foraging success and strike accuracy differed between albino and pigmented toads, and foraging success differed among light treatments for both albinos and pigmented individuals. Albinos consumed fewer termites than did pigmented individuals in both bright and dim light, and their foraging success was reduced in dim light relative to bright light, whereas pigmented toads had similarly high foraging success in bright and dim light. This result suggests that albinos require more light than do pigmented toads to forage successfully, which probably has a strong effect on competitive ability *in situ* because toads are primarily nocturnal [92,93]. Poor visual acuity might also encourage shifts towards diurnal activity in albinos, rendering them more susceptible to desiccation, ultraviolet radiation and predation. Both albino and pigmented toads consumed few termites in total darkness, confirming toads’ reliance on vision while foraging. During observation trials, albinos had lower strike accuracy than did pigmented toads, probably due to impairment of stereoscopic vision [64]. Albinos struck less successfully than did pigmented individuals, probably using more energy to forage than their more accurate pigmented siblings. Although the potential for off-target effects of CRISPR modification cannot be completely ignored, the results of our behaviour trials suggest that the effects of albinism on visual acuity and stereoscopic vision played a causal role in the reduced survival and competitive ability of albinos relative to pigmented toads. Some albino toads probably could not forage successfully enough to survive, even without competition from pigmented individuals. The albinos that did survive required brighter conditions and higher energy expenditures when foraging than did pigmented toads, placing albinos at a competitive disadvantage that manifested in reduced growth rates.

Our competition experiments and behavioural trials suggest that albinism is probably selected against in the wild for reasons other than or in addition to increased susceptibility to predation. Although we did not test effects of predation in these experiments and it probably contributes to selection against albinism [53–55,57], our results indicate that competitive inferiority also results in selection against albinism. This appears to be especially true for visual predators, in which visual impairment reduces foraging success in albinos relative to pigmented individuals. However, the lowered viability of albino tadpoles suggests that albino vertebrates that do not rely on vision to forage (tadpoles rely heavily on chemical cues [69]) may also be at a competitive disadvantage. Although we provide strong evidence for associations between visual impairment, competitive inferiority and reduced fitness, effects of albinism on other aspects of physiology (e.g. susceptibility to UV radiation and immune function [48,66,67]) and behaviour [60,61,72,86,87] may also play a role in selection against albinos in the wild. Further experiments using gene knockouts should be conducted to assess how these factors interact to suppress albinism in wild populations.

Without CRISPR knockouts, the powerful sibling-to-sibling comparison that we used to draw these conclusions would have been difficult to achieve, requiring capture of naturally occurring albinos and several generations of breeding. Sibling versus sibling comparisons of the effects of other phenotypic traits may not be possible with selective breeding, especially if the focal trait does not conform to simple Mendelian inheritance. The alternative, breeding strains for specific traits, introduces confounding kinship effects, weakening such comparisons. CRISPR knockouts solve these problems, allowing for cost-effective, robust tests of the functional and fitness consequences of phenotypic traits. Knockouts are widely used in medical research, immunology and developmental biology [8,94,95], and our work illustrates their utility for ecologists and evolutionary biologists. By knocking out specific genes, researchers can now test longstanding theories about the fitness consequences of phenotypes and their relationship to ecological context. Why are certain traits favoured or eliminated by selection? Why are polymorphisms maintained in wild populations? What drives local adaptation and what limits it? Gene knockouts offer powerful tools to answer these questions, and because knockouts are not considered genetically modified organisms in some jurisdictions, we can potentially run such experiments in the field as well as in the laboratory. Where permissible, knockouts could be used for *in situ* manipulative experiments assessing the fitness consequences of phenotypic variation in different environments. This approach represents a transformative step towards understanding the ecological and evolutionary consequences of trait variation in nature.

## Data Availability

All data presented in this manuscript and the code used to analyse those data are available as supplementary material [96].
